# Influence of High-Temperature Oxidation and Test Conditions on the Dynamic Mechanical Properties of 2.5D SiC_f_/SiC_m_ Composites

**DOI:** 10.3390/ma14010145

**Published:** 2020-12-31

**Authors:** Chenxi Yang, Jiejie Wu, Allah Ditta, Sujun Wu, Zihua Zhao

**Affiliations:** 1 School of Materials Science and Engineering, Beihang University, Beijing 100191, China; yangcxyoung@buaa.edu.cn (C.Y.); allahditta@buaa.edu.cn (A.D.); 2 School of Energy and Power Engineering, Beihang University, Beijing 100191, China; wujiesilence@163.com

**Keywords:** SiC_f_/SiC_m_ composites, heat treatment, oxidation, storage modulus, internal friction

## Abstract

The influence of microstructure evolution on the dynamic mechanical properties, including storage modulus and internal friction, of the 2.5D SiC_f_/SiC_m_ composites after high-temperature treatment (800 °C and 1400 °C) in the air was investigated by three-point bending vibration test. The effects of test frequency and amplitude on storage modulus and internal friction were also evaluated. The results show that as-prepared samples have maximum storage modulus and internal friction. However, the composites treated at 800 °C in the air have the minimum storage modulus due to a large number of defects produced within the composite structure, and the composites treated at 1400 °C have the minimum internal friction due to the formation of α-cristobalite in the interface between the matrix and fibers, resulting in stronger interface bonding. With regard to test conditions, the storage modulus is sensitive to amplitude but not frequency; however, the internal friction is sensitive to both frequency because of anelasticity and amplitude due to the static hysteresis.

## 1. Introduction

Turbine engines suffer severe dynamic loads caused by structural vibration and noise during service, and these vibrations should not exceed their resonance limit, so as not to damage the engine [1,2]. The dynamic mechanical properties such as storage modulus and internal friction are therefore of significant importance in order to ensure the safe service of the turbine engines. Internal friction (i.e., damping) is regarded as one of the most important dynamic mechanical properties and refers to the conversion of vibration energy into internal energy during mechanical vibration. It can also be used as a non-destructive testing method to study material microstructures due to the sensitivity to the variation of microstructure [3,4,5].

The continuous silicon carbide fiber-reinforced silicon carbide ceramic matrix composite (SiC_f_/SiC_m_) is considered to be an ideal structural material in turbine engines due to its light weight and large resistance to aggressive environments [6,7]. The SiC_f_/SiC_m_ composite is composed of SiC fibers, SiC matrix and interface layer phase between fiber and matrix. The interfacial phase is usually pyrocarbon (PyC).

The internal friction of SiC_f_/SiC_m_ composites has received increasing interest, and some valuable results have been obtained. The research by Sato et al. [8] showed that SiC_f_/SiC_m_ composites fabricated by the chemical vapor infiltration method exhibited suitable internal friction and elastic modules compared to the methods of the polymer impregnation and pyrolysis and the hot press due to the different matrix microstructure. Similarly, Hou et al. [1,9] presented that different woven structure could produce different internal friction; for example, the 3D 5-directional SiC_f_/SiC_m_ composites had smaller internal friction than the 3D 4-directional SiC_f_/SiC_m_ composites. In addition, the composites with an interphase layer also showed lower internal friction than those without an interphase layer. SiC_f_/SiC_m_ composites will face high-temperature oxidation environment and bear the dynamic load as turbine engine parts, and the influence of structural evolution of composite materials on dynamic mechanical properties is very important for the safe use of engines in high-temperature oxidation environment; however, the relevant research is very limited in the literature.

It has been shown that the internal friction test can be used as a non-destructive testing method to study the evolution of material microstructures and effectively assess the damping performance of materials. In this work, therefore, the storage modulus and internal friction behavior of 2.5D SiC_f_/SiC_m_ composites with a single layer of PyC treated at high temperature in the air atmosphere are investigated by internal friction test, and the corresponding mechanisms are discussed in details. The influence of varying test conditions is also evaluated.

## 2. Materials and Methods

**Figure 1:**
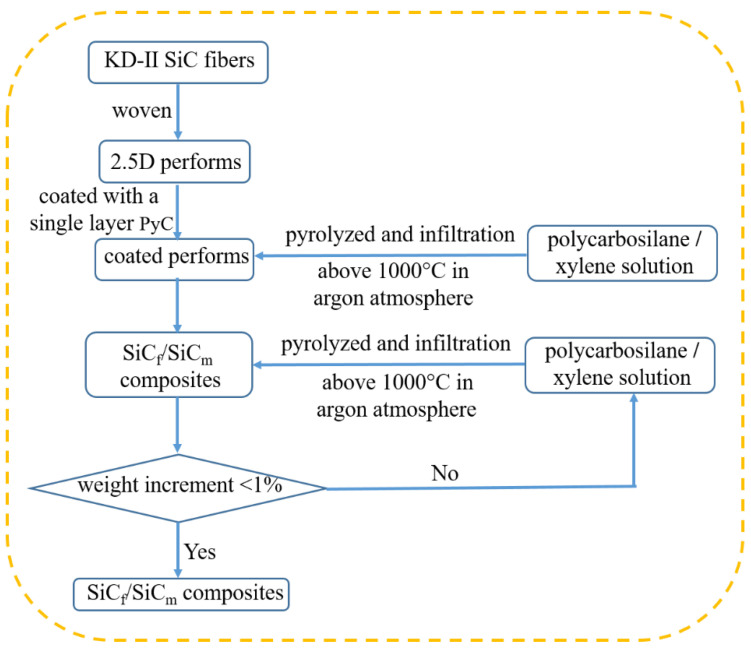
Fabrication flowchart of the composite.

The 2.5D SiC fiber preforms were woven with KD-II SiC fiber bundles and then coated with a single layer of PyC using the chemical vapor deposition method. The polycarbosilane/xylene solution, as the precursor, was utilized to infiltrate the coated performs in vacuum condition and subsequently pyrolyzed above 1000 °C to fabricate the SiC_f_/SiC_m_ composites in an argon atmosphere. The process of infiltration and pyrolysis was repeated until the weight increment was below 1% to finish the fabrication. The fabrication flowchart of the composite is shown in Figure 1. Some of the as-prepared composites (named as A-P) were then oxidized in air at 800 °C and 1400 °C, henceforth denoted as A-800 and A-1400, respectively, for 1h in a muffle furnace.

The surface morphologies of the as-prepared and high-temperature-oxidized SiC_f_/SiC_m_ composites were observed by field emission scanning electron microscopy (FESEM, JSM-7001F, JEOL Ltd.,Tokyo, Japan), and the phase constituent was studied by X-ray diffraction (XRD, D/MAX-2500, Rigaku, Tokyo, Japan) analysis. The thermal behavior of SiC_f_/SiC_m_ composites was analyzed from room temperature to 1500 °C at a heating rate of 10 °C/min under air atmospheres by thermogravimetric and differential scanning calorimetry analysis (TG-DSC, STA-449F3, NETZSCH, Selb, Germany).

The internal friction tests were performed under three-point bending vibration at room temperature using a dynamic mechanical analyzer (DMA, Q800, TA Instruments, New Castle, DE, USA) with a sample size of 20 mm × 3 mm × 1.5 mm. The test conditions were as follows: (1) the frequency ranged from 1 to 50 Hz at the constant strain amplitude of 10 μm; (2) the strain amplitude ranged from 1 to 11 μm at the constant frequency of 10 Hz.

## 3. Results and Discussion

### 3.1. Evolution of Microstructure

**Figure 2:**
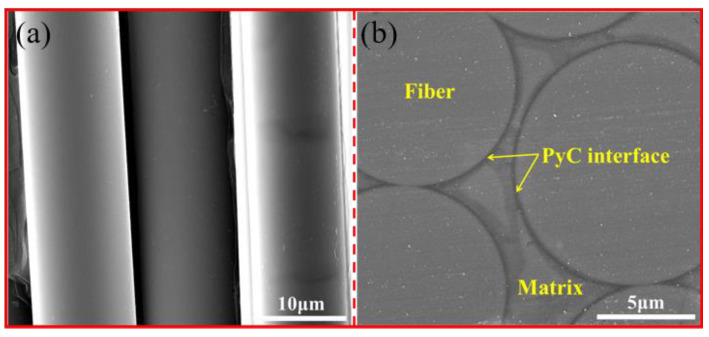
Microstructure morphologies of the as-prepared SiCf/SiCm composites. (**a**) warp direction and (**b**) weft direction.

**Figure 3:**
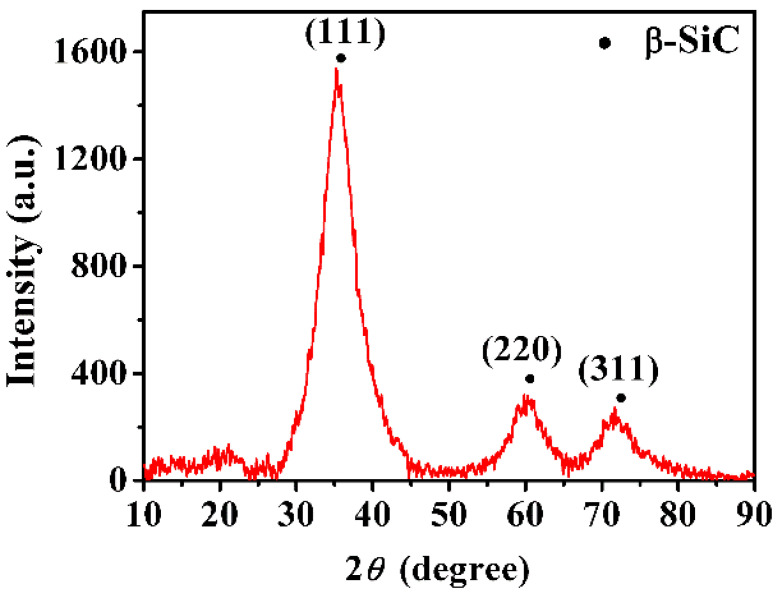
XRD pattern of the as-prepared SiC_f_/SiC_m_ composites.

Figure 2 shows the surface morphology of the A-P composite. The fibers coated with a single layer of PyC present a smooth surface as shown in Figure 2a. The fibers, matrix, and PyC interface layers can be clearly identified, and the morphologies of these components are completely intact, as evident in Figure 2b. The XRD pattern of the A-P composite is presented in Figure 3. The result reveals that three peaks correspond to (111), (220), and (311) planes of β-SiC, which indicate that the SiC phase in fibers and matrix belongs to β-SiC.

**Figure 4:**
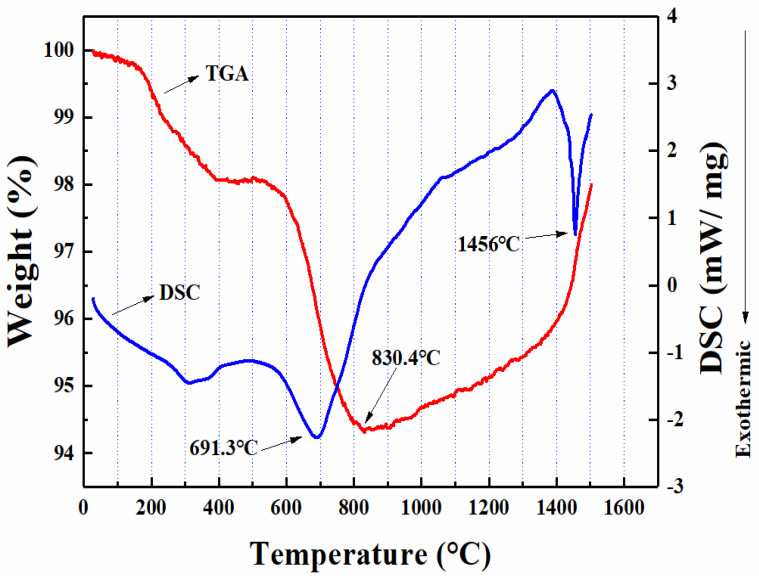
TGA-DSC curves of SiC_f_/SiC_m_ composites from room temperature to 1500 °C.

Figure 4 shows the thermal behavior of SiC_f_/SiC_m_ composite. From room temperature to 500 °C, the weight of the composite decreased by about 1.91% due to oxidation and combustion of some low-melting impurities, which could have been induced during fabrication process. In the temperature stage of 500–830.4 °C, the weight of the composite continued to decrease by 3.79%. Meanwhile, the exothermal peak appeared in the DSC curve at 691.3 °C, indicating that the decrease in the weight was due to the oxidation of some substances. When the temperature exceeded 830.4 °C, the weight began to increase, and the weight increased by 1.66% at 1400 °C. At the stage of 1400–1500 °C, the weight increased by 2.04% in total, and the DSC curve showed another exothermal peak at 1456 °C, indicating that a large amount of oxidation occurred at this stage to increase the weight of the composite. The SiC_f_/SiC_m_ composite was employed as flame-holder and exhaust cone materials in a turbine engine and required to be compatible with the minimum operating temperature of 800 °C. Similarly, the SiC_f_/SiC_m_ composite as the turbine inlet in modern industrial turbines will withstand temperatures in excess of 1300 °C [10,11,12,13]. Considering the application conditions, 800 °C and 1400 °C were therefore selected for subsequent oxidation treatment of the composite.

**Figure 5:**
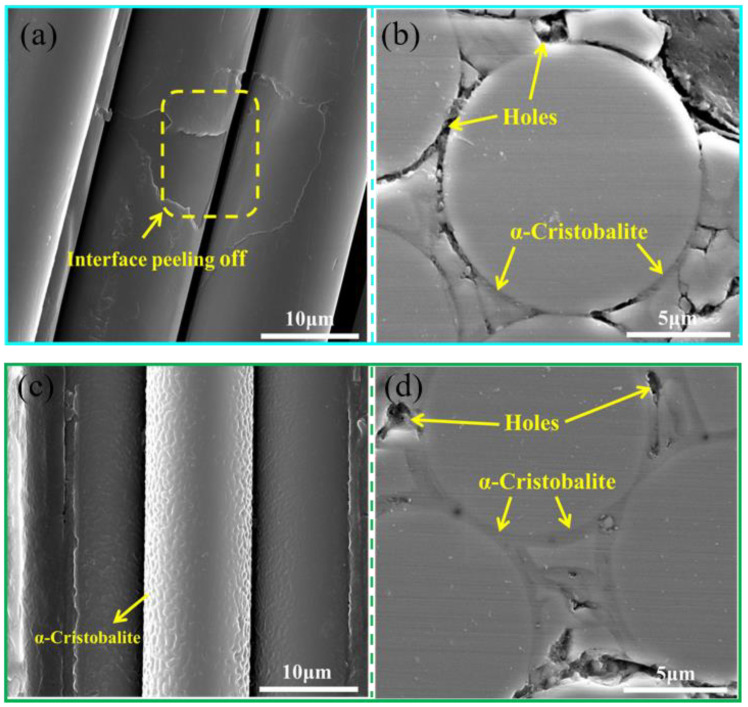
Microstructure morphologies of the 2.5D SiC_f_/SiC_m_ composites under different treatment conditions. (**a**,**b**) A-800, (**c**,**d**) A-1400.

Figure 5 shows the surface morphologies of the SiC_f_/SiC_m_ composites, heat-treated at 800 °C and 1400 °C for 1h, in air atmosphere. The PyC layers partially peeled off from the fiber surface following heat treatment at 800 °C (Figure 5a), and some holes also developed at the interface, as shown in Figure 5b. It can therefore be suggested that interface oxidation occurred when the composite was heated at 800 °C in air, resulting in interface debonding and peeling off in A-800 composite. Generally, PyC is prone to oxidize at temperatures as low as 500 °C in air atmosphere [14], leading to the weight loss of the composite, which is consistent with the TG-DSC results in the range of 500–800 °C. It was notable that few α-cristobalite phases can be observed at the interface between fibers and matrix. SiC_f_/SiC_m_ composites are likely prone to passive oxidation under high temperatures and high partial pressures of oxygen. The oxidation process of SiC_f_/SiC_m_ composites is represented in terms of the following expressions [15]:

(1) $$C_{\left(s\right)}+O_{2\left(g\right)}\to CO_{2\left(g\right)}$$

(2) $$SiC_{\left(s\right)}+2O_{2\left(g\right)}\to SiO_{2\left(s\right)}+CO_{2\left(g\right)}$$

With the increase in temperature, the composite went through severe oxidation at 1400 °C. The surface of fibers was completely covered by α-cristobalite and presented a coarse appearance, as shown in Figure 5c. The interphase between the fiber and matrix was filled with the α-cristobalite phase, and a few holes were still found in the composites (Figure 5d). Due to the formation of α-cristobalite, the weight of the composite increased, which is consistent with the TG-DSC results in the range of 800–1500 °C.

**Figure 6:**
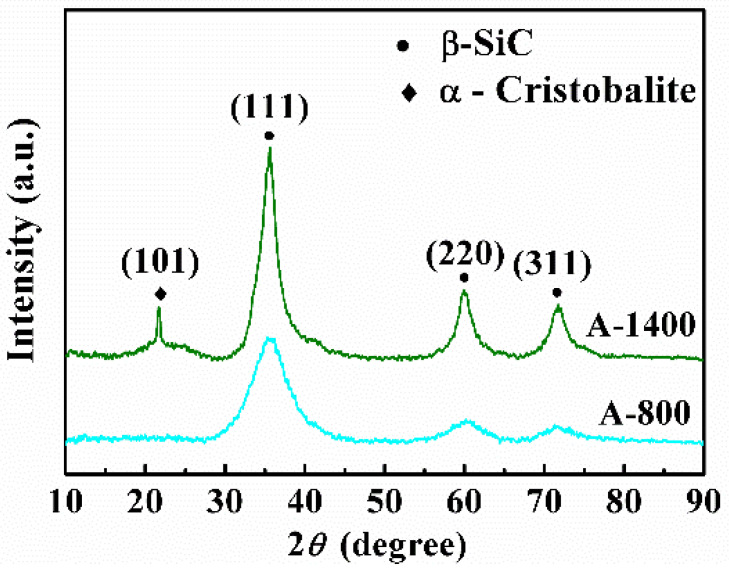
XRD patterns of the 2.5D SiCf/SiCm composites under different treatment conditions.

Figure 6 shows the XRD patterns of the composites heat-treated in air atmospheres at 800 °C and 1400 °C. The β-SiC can still be observed as the main phase at both temperatures. A strong peak around a 2θ value of 21.8° for A-1400 composite verifies the presence of α-cristobalite; however, no prominent diffraction peak of α-cristobalite is observed for A-800 composite, probably because the amount of α-cristobalite is too little to detect by XRD.

### 3.2. The Influence of Microstructure and Test Conditions on the Storage Modulus

**Figure 7:**
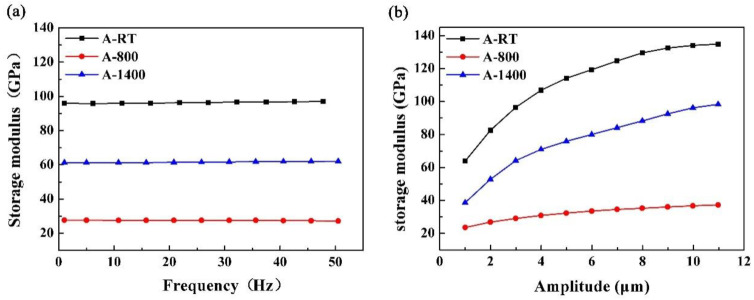
Plots of the storage modulus of the composites versus frequency (**a**) and amplitude (**b**).

It can be seen from Figure 7a,b that the A-P composite has the highest storage modulus, while the A-800 composite has the lowest storage modulus with the increase in frequency and strain amplitude. During heat treatment at 1400 °C in air, the β-SiC was severely oxidized to α-cristobalite, and the PyC was also replaced by α-cristobalite at the interface. The moduli of these three substances are ordered as follows: E_β-SiC_ (460 GPa in pore-free state [7]) > E_α-cristobalite_ (59.4 GPa [16]) > E_PyC_ (30.2 GPa [17]). According to the mixing law, the storage modulus relates to the volume of the constituents in the composites. Due to the formation of large amounts of α-cristobalite, the modulus of A-1400 sample was reduced as compared to the A-P sample. In addition, the modulus of the material is also strongly dependent on microstructure defects. Due to the consumption of PyC layer, a large number of defects could have formed at the initial stage of oxidation in A-1400 samples. Subsequently, the appearance of α-cristobalite could heal some defects, but the total amount of defects in A-1400 samples are still high (Figure 5), which would decrease the storage modulus of the A-1400 sample. Likewise, the A-800 sample has the most defects, resulting in the lowest storage modulus.

The storage modulus of the A-P, A-800, and A-1400 samples remain nearly constant from 1 to 50 Hz in testing frequency, indicating that the frequency has no obvious effect on the storage modulus of the composites. However, the storage modulus increases monotonically with the testing strain amplitude in the range of 1 to 11 μm. The storage modulus represents the ability to store elastic deformation energy of composites. In the range of elastic deformation, the stress increases with displacement (strain amplitude), and the energy stored in the composite increases with the displacement. In addition, the increase in stress may also reduce the microstructure defects through stress-induced orientation [9]. Therefore, the storage modulus of all composites increases with the strain amplitude.

### 3.3. The Influence of Microstructure and Test Conditions on the Internal Friction

**Figure 8:**
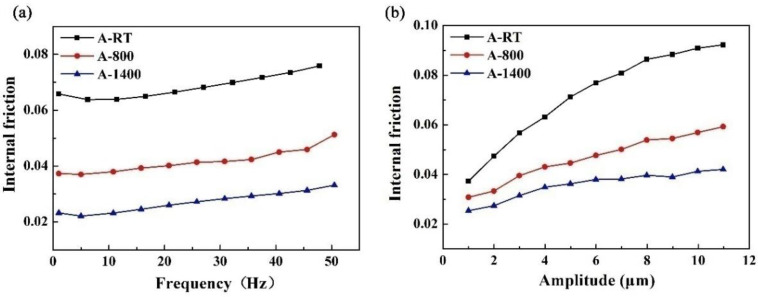
Plots of the internal friction of the composites versus frequency (**a**) and amplitude (**b**).

The internal friction (*Q*^−1^) was calculated according to the following equation [18]:

(3) $$Q^{-1}=tan\delta =\frac{E^{”}}{E’}$$

where *δ* is the loss angle between applied stress and strain, *E′* is the storage modulus and *E″* is the loss modulus. Experimental results show that the A-P composite has the highest internal friction values, while the A-1400 composite has the lowest values with the change in frequency and amplitude (Figure 8). Generally, defects such as dislocations, holes, and cracks, are considered as the source of internal friction in composites. Moreover, the existence of interfaces between fiber and matrix may influence the internal friction. The weak interface bonding strength can easily cause interface slip, which could consume energy. In the A-P samples, the existence of PyC interface as well as defects such as holes, dislocations, and cracks, lead to the high internal friction. For the A-800 samples, the oxidation of PyC at the interface could increase the interface friction. However, the interface bonding could strengthen due to the presence of α-cristobalite at the interface, making interface slip difficult [19], which thus leads to the reduction of internal friction. Some dislocations could also be eliminated in the process of heat treatment [20], reducing the source of internal friction. Consequently, the internal friction of A-800 samples is lower than that of A-P samples. In the A-1400 samples, more PyC was replaced by α-cristobalite through oxidation, resulting in a stronger interface bonding. At the same time, more dislocations would be eliminated during the high-temperature heat treatment; hence, the A-1400 composite has the minimum internal friction compared to A-P and A-800 samples.

It is noteworthy that the internal friction increases with frequency in the range of 1 to 50 Hz (Figure 8a). The internal friction includes the anelasticity internal friction and static hysteresis. The former is strongly dependent on frequency, and the latter is relevant to the amplitude [21]. The anelasticity internal friction may be induced between the interface of elastic material and viscous material and enhanced with increasing testing frequency due to the deformation inharmony of constituents at a certain frequency [22]. Generally, the PyC could be regarded as a viscous material, whereas SiC matrix and SiC fibers are elastic, which causes the interface slip, resulting in an increase in internal friction with frequency for A-P sample. It can be seen from Figure 8b that the internal friction of A-800 and A-1400 samples also rise with the increase of frequency, which indicates that PyC still exists in these composites and was not completely oxidized. Moreover, the composites are vigorously vibrated under flexural stress in the tests, which could generate cyclic heat flow from the region of compressive stress to the region of tensile stress in these inhomogeneous composites, resulting in the thermoelastic internal friction first postulated by Zener [23]. According to Zener, the relationship of Equation (4) indicates that thermoelastic internal friction increases with frequency.

(4) $$Q^{-1}=tan\delta =\frac{E_{u}\alpha^{2}T_{0}}{C_{\sigma }}\frac{\omega \tau }{1+\left(\omega \tau^{2}\right)}$$

where *τ* = (*C_σ_*a^2^)/(π^2^k_th_), a is the sample thickness, k_th_ is the thermal conductivity, *C_σ_* is the specific heat per unit volume at constant stress, α is the coefficient of thermal expansion, *T*_0_ is the absolute temperature, and *ω* is the angular frequency.

The internal friction increases with the increase in strain amplitude in the range of 1 to 11 μm, as observed in Figure 8b, which is related to static hysteresis dependence on amplitude. During the three-point bending forced vibration tests, the stress–displacement curves of composites display the hysteresis loop under cyclic stress [24]. The area of the hysteresis loop corresponds to the internal dissipated energy of the composite and increases with strain amplitude.

As described above, internal friction depends on the composition and microstructure of the material. The microstructure evolution of SiC_f_/SiC_m_ composites under a high-temperature oxidation environment will lead to a reduction in internal friction. Generally, if the internal friction is too small, it will not be enough to dissipate sufficient energy, which may cause resonance to damage the turbine engines [25]. Therefore, the oxidation tendency of SiC_f_/SiC_m_ composites must be limited. At present, adding environmental barrier coating on the surface of composites is a feasible method to isolate the composite from the external environment [26,27]. In addition, improving the fabrication process and increasing the density of the SiC_f_/SiC_m_ composites to reduce the oxygen diffusion channel into the composites can also help to improve the internal friction performance.

The increase in frequency and amplitude are conducive to increasing the internal friction, as mentioned above. It should be pointed out that too high frequency and amplitude will also cause resonance of the material, leading to cyclic fatigue failure [28,29]. Therefore, frequency and amplitude must be limited to a safe range. Moreover, under high frequency and high amplitude load, the internal friction capacity of the material itself is not enough to dissipate enough energy, and the damper must be used to reduce the vibration of the material to ensure the safe use of the turbine engines.

## 4. Conclusions

The heat treatment significantly affected the composition and microstructure of 2.5D SiC_f_/SiC_m_ composites. After treatment at 800 °C for 1 h, partial peeling off of the interface and a few α-cristobalite phases were observed on the surface of the composite. After treatment at 1400 °C for 1 h, the surface of the fibers was severely oxidized and the PyC interface was replaced by α-cristobalite from the passive oxidation of SiC, leading to the strongest bonding of fibers and matrix.

The A-P samples had maximum storage modulus and internal friction. The A-800 samples displayed minimum storage modulus due to the presence of relatively more defects. The A-1400 samples possessed minimum internal friction due to the formation of α-cristobalite at the interface. The storage modulus was sensitive to amplitude only; however, the internal friction was not only sensitive to frequency due to the anelasticity internal friction and thermoelastic internal friction, but also to amplitude due to the static hysteresis.

## Data Availability

Data sharing is not applicable to this article.

## References

[1] Hou Z.H., Luo R.Y., Yang W., Xu H.Z., Han T. (2016). Effect of fiber directionality on the static and dynamic mechanical properties of 3D SiCf/SiCm composites. Mater. Sci. Eng. A.

[2] Elsaid A., Jamiy F.E., Higgins J., Wild B., Desell T. (2018). Optimizing long short-term memory recurrent neural networks using ant colony optimization to predict turbine engine vibration. Appl. Soft. Comput..

[3] Ide N., Yamashita M., Asano S. (1999). Solid solution hardening evaluated from amplitude-dependent internal friction in polycrystalline copper alloys. Scr. Mater..

[4] Ouytsel K.V., Batist R.D., Schaller R. (2000). Dislocation-defect interactions in nuclear reactor pressure-vessel steels investigated by means of internal friction. J. Alloys Compd..

[5] Jia Y., Li K.Z., Xue L.Z., Ren J.J., Zhang S.Y. (2017). Internal friction behaviour of carbon fibre reinforced multilayered (PyC-SiC)_n_ matrix composites. Compos. Part B.

[6] Padture N.P. (2016). Advanced structural ceramics in aerospace propulsion. Nat. Mater..

[7] Lamon J., Konings R. (2012). Properties and Characteristics of SiC and SiC/SiC Composites. Comprehensive Nuclear Materials.

[8] Sato S., Serizawa H., Araki H., Noda T., Kohyama A. (2003). Temperature dependence of internal friction and elastic modulus of SiC/SiC composites. J. Alloys Compd..

[9] Hou Z.H., Luo R.Y., Yang W., Xu H.Z., Han T. (2016). Effect of interface type on the static and dynamic mechanical properties of 3D braided SiCf/SiCm composites. Mater. Sci. Eng. A.

[10] Spriet P., Habarou G. (1997). Application of CMCs to Turbojet Engines: Overview of the SEP Experience. Key Eng. Mater..

[11] Naslain R. (2004). Design, preparation and properties of non-oxide CMCs for application in engines and nuclear reactors: An overview. Compos. Sci. Technol..

[12] Konter M., Thumann M. (2001). Materials and manufacturing of advanced industrial gas turbine components. J. Mater. Process Technol..

[13] Murthy P.L.N., Nemeth N.N., Brewer D.N., Mital S. (2008). Probabilistic analysis of a SiC/SiC ceramic matrix composite turbine vane. Compos. Part B.

[14] Naslain R., Pailler R., Lamon J. (2010). Single- and Multilayered Interphases in SiC/SiC Composites Exposed to Severe Environmental Conditions: An Overview. Int. J. Appl. Ceram. Technol..

[15] Fitzgerald K., Shepherd D. (2018). Review of SiCf/SiCm m, corrosion, erosion and erosion-corrosion in high temperature helium relevant to GFR conditions. J. Nucl. Mater..

[16] Yeganeh-Haeri A., Weidner D.J., Parise J.B. (1992). Elasticity of α-cristobalite: A silicon dioxide with a negative Poisson’s ratio. Science.

[17] Gross T.S., Nguyen K., Buck M., Timoshchuk N., Tsukrov I.I., Reznik B., Piat R., Böhlke T. (2011). Tension–compression anisotropy of in-plane elastic modulus for pyrolytic carbon. Carbon.

[18] Menard K.P. (2008). Dynamic Mechanical Analysis: A Practical Introduction.

[19] Becher P.F., Lin H.T., More K.L. (1998). Lifetime-applied stress response in air of a SiC-based Nicalon-fiber-reinforced composite with a carbon interfacial layer: Effects of temperature (300 °C to 1150 °C). J. Am. Ceram. Soc..

[20] Wang F.Y., Cheng L.F., Liang S.H. (2019). Study on the internal friction mechanism of C/SiC composites in different corrosion stage. Vacuum.

[21] Hou J.T., Qiao S.R., Lu G.F., Zhang C.Y., Zhang Y.B. (2007). Internal friction of a 2D-C/SiC composite from 25 °C to 400 °C. Key Eng. Mater..

[22] Hou J.T., Qiao S.R., Lu G.F., Zhang C.Y., Zhang Y.B. (2009). Influence of heat treatment on the internal friction of 2D-C/SiC composites. J. Mater. Process. Technol..

[23] Zener C. (1948). Elasticity and Anelasticity of Metals.

[24] Granato A., Lücke K. (1956). Theory of mechanical damping due to dislocations. J. Appl. Phys..

[25] Tereshchenko Y.M., Doroshenko E.V., Tehrani A., Abolhassanzade J. (2015). Aerodynamic factors of influence on the resonance vibration of gas turbine compressor blades. Strength Mater..

[26] Ramasamy S., Tewari S.N., Lee K.N., Bhatt R.T., Fox D.S. (2011). Mullite–gadolinium silicate environmental barrier coatings for melt infiltrated SiC/SiC composites. Surf. Coat. Technol..

[27] Lv B., Jin X., Cao J., Xua B., Wang Y., Fang D. (2020). Advances in numerical modeling of environmental barrier coating systems for gas turbines. J. Eur. Ceram. Soc..

[28] Berthelot J.M., Assarar M., Sefrani Y., Mahi A.E. (2008). Damping analysis of composite materials and structures. Compos. Struct..

[29] Griffin J.H. (1990). A Review of Friction Damping of Turbine Blade Vibration. Int. J. Turbo Jet Engines.

